# High adherence to the Mediterranean diet and Alternative Healthy Eating Index are associated with reduced odds of metabolic syndrome and its components in participants of the ORISCAV-LUX2 study

**DOI:** 10.3389/fnut.2022.1087985

**Published:** 2022-12-13

**Authors:** Kinda Al Kudsee, Farhad Vahid, Torsten Bohn

**Affiliations:** Nutrition and Health Research Group, Department of Precision Health, Luxembourg Institute of Health, Strassen, Luxembourg

**Keywords:** inflammation, oxidative stress, heart disease, type 2 diabetes, hypertension, dietary indices

## Abstract

**Background:**

Metabolic syndrome (MetS) is a major risk factor for cardiometabolic complications. Certain dietary patterns play a pivotal role in improving MetS components. The aim of this investigation was to study associations between the Mediterranean Diet Score (MDS) and the Alternative Healthy Eating Index (AHEI) and the odds of MetS and its components in adults living in Luxembourg.

**Methods:**

Data from 1,404 adults participating in the cross-sectional ORISCAV-LUX2 study were analyzed by a self-reported questionnaire, anthropometric measures, a food frequency questionnaire (174 items), and blood/urine samples.

**Results:**

A significant association of dietary indices and MetS was not found except when expressing MetS as a score (continuous variable, log-transformed), based on the weighting of compounds using exploratory factor analysis with the MDS (β = −0.118, 95% CI: −0.346, −0.120) and AHEI (β = −0.133, 95% CI: −0.059, −0.019). Fully adjusted linear regression models further showed significant inverse associations between components of MetS and MDS (all as log-transformed variables), including body mass index (BMI) (β = −0.0067, 95% CI: −0.0099, −0.0036), waist-circumference (WC) (β = −0.0048, 95% CI: −0.0072, −0.0024), systolic blood pressure (SBP) (β = −0.0038, 95% CI: −0.0061, −0.0016), and diastolic blood pressure (DBP) (β = −0.0035, 95% CI: −0.0060, −0.0009). Similarly, significant inverse associations between AHEI and components of MetS (log-transformed) included BMI (β = −0.0001, 95% CI: −0.0016, −0.0002), WC (β = −0.0007, 95% CI: −0.0011, −0.0002), SBP (β = −0.0006, 95% CI: −0.0010, −0.0002), and DBP (β = −0.0006, 95% CI: −0.0011, −0.0001).

**Conclusion:**

Higher adherence to a Mediterranean diet and following healthy eating guidelines were associated with reduced odds of MetS and several of its components in Luxembourgish residents, highlighting that balanced and healthy eating patterns are a crucial cornerstone in the fight against MetS.

## Introduction

Metabolic syndrome (MetS) is a metabolic and public health-related condition that burdens the life of people and also the healthcare system globally. It is a major risk factor for cardiometabolic complications, affecting over a billion people worldwide (1), with a prevalence of 28% in adults in Luxembourg (2). MetS is a multi-faceted disorder characterized by a cluster of interrelated risk factors for coronary heart disease (CHD), cardiovascular diseases (CVD), and type 2 diabetes (T2D) (1). There are various definitions and diagnostic criteria for MetS. The most generally used and documented criteria are based on the National Cholesterol Education Program Adult Treatment Panel III (NCEP ATP III) (3). MetS is diagnosed when three or more of the following five main components are fulfilled: dyslipidemia, elevated arterial blood pressure (BP), dysregulated glucose homeostasis, abdominal obesity, and/or insulin resistance (4).

One of the most important aspects of MetS is that it aids in screening individuals at high risk of developing CVD, T2D, hypertension, and atherosclerosis, together with increased disease mortality (5). MetS is linked to a 1.5-fold increase in all-cause mortality and a 2-fold elevation of CVD risk (6). In order to decrease the risk of CVD, T2D, and other complications, drug therapy for metabolic components is considered in some cases (7). However, early prevention measures for this condition are more effective when employed on a population-wide scale rather than drug therapy treatment. Nutrition is among the most significant modifying lifestyle factors influencing cardio-metabolic health (8). Modest weight reduction by lifestyle modification, including a healthy diet and sufficient physical activity, are a practical and effective means that can improve all five components of MetS, as lifestyle modification controls the entire metabolic profile of individuals with MetS and selectively mobilizes the abdominal visceral fat (9, 10).

Despite the widespread occurrence and significance of MetS, there is no consensus about the contribution of individual dietary risk factors to MetS. However, several meta-analyses investigating risk factors for MetS have shown that a healthy diet can significantly reduce the (pooled relative) risk of developing MetS (by 15%) and CVD (by 31%) (8, 11, 12). For example, it has been shown that dietary patterns such as a diet low in saturated fat (13), low in simple sugars (14), and reduced meat consumption (15) but high in fruits and vegetables (16) can reduce the risk of MetS. However, rather than focusing only on individual dietary aspects, more integrative and comprehensive approaches that investigate the entirety of food items consumed are sought that characterize dietary properties in a more holistic approach. Such approaches have the primary goal of synthesizing a significant quantity of dietary data (a food item or a nutrient) into an overall score that can be used to evaluate potential risk factors or relate to a health outcome. Two such prominent indices include the Alternative Healthy Eating Index (AHEI) and the Mediterranean Diet Score (MDS).

The AHEI is an index developed by researchers at the Harvard School of Public Health as an alternative measure of diet quality, focused on absolute food intake rather than nutrient density, to identify the future risk of diet-related chronic disease. The AHEI is a valuable tool that measures adherence to dietary guidelines and evidence-based recommendations for nine food groups or aspects, including vegetables, fruits, nuts, and soy protein, the ratio of white to red meat, fiber, trans-fatty acids, the ratio of PUFA/SFA, multivitamin supplement intake, and alcohol (17). A multiethnic Cohort found that better diet quality—as assessed by AHEI, was associated with an 18–26% lower risk of all-cause and CVD mortality; meta-analyses also confirmed these results (18, 19). Several mechanisms for how a higher AHEI may relate to cardiometabolic diseases have been described, such as the direct effects of fibers in reducing cholesterol absorption, the impact of polyphenols in reducing oxidative stress and inflammation, as well as their indirect effects through gut microbiota (20). The MDS is based on solid epidemiologic evidence and evaluates adherence to the Mediterranean diet (MD) based on the consumption of selected foods, especially considering fish intake in addition to legumes, vegetables, fruits, and grains as an advantageous group, and dairy products, red meat, lipids, and alcohol as an adverse health food group (21). High MDS (indicating adherence to an MD) is characterized by high consumption of fish and seafood, olive oil as the primary fat, vegetables, fruits, whole grains, legumes, and nuts, with a high MUFA/SFA ratio; and low consumption of red meat and meat products is related to a lower risk of cardiometabolic diseases. The high amounts of unsaturated fats (MUFA and PUFA), fiber, and polyphenols in this diet and other factors, such as the low intake of SFA, contribute to its beneficial effects in reducing the risk of such diseases, including MetS (22). In addition, recently, the regulatory role of nutritional components of the MD on the gut microbiota and the immune system and the relation to non-communicable diseases, including obesity, T2D, CVD, MetS, and some types of cancer, has been emphasized (23). Evidence suggests that MD adherence was able to modulate the gut microbiota and increases its diversity, which has been related to increased short-chain fatty acid (SCFA) production, among others (24).

It has been shown that assessing overall diet quality, rather than specific nutrients or food components, is more successful in predicting a relationship between diet and disease (25). According to our knowledge, no previous study has used MDS and AHEI together for estimating the risk of MetS. Since these two indicators somehow complement each other (in terms of components) and surveys using them together are limited, a combined investigation was felt appropriate. In addition, no association between dietary indices such as MDS and AHEI with MetS and its components among residents of Luxembourg, a country characterized by a high rate of risk factors such as obesity and high blood pressure (26) and quite diverse dietary habits/culinary landscape (due its almost 50% foreign residents), has been reported on thus far. Therefore, it is important to shed light on this area with the aim of better controlling MetS. This study strives to derive a more recent estimation of this association by determining the relation between MDS, AHEI score, and MetS and its components among residents of Luxembourg.

## Materials and methods

### Study protocol and design

This analysis is based on the national survey “Observation of Cardiovascular Risk Factor in Luxembourg (ORISCAV-LUX 2),” which is the second wave of a cross-sectional study, carried out between January 2016 and January 2018 to assess risk factors of CVD in the Luxembourgish adult population (27). In the original ORISCAV-LUX survey (2007–2008), *n* = 1,432 participants were included by a systematic random sampling procedure. In the following ORISCAV-LUX 2 survey, participants were retained by an initial baseline and complimentary sampling. A total of 660 individuals participated in both studies. In the present analysis, the participants were randomly selected based on sociodemographic attributes, including the district of residence, age, and gender. A full description of the sampling procedure in the first and second waves has already been published elsewhere (28). This study was approved by the National Research Ethics Committee (CNER, No 201-505/12) and the National Commission for Data Protection (CNPD).

**FIGURE 1:**
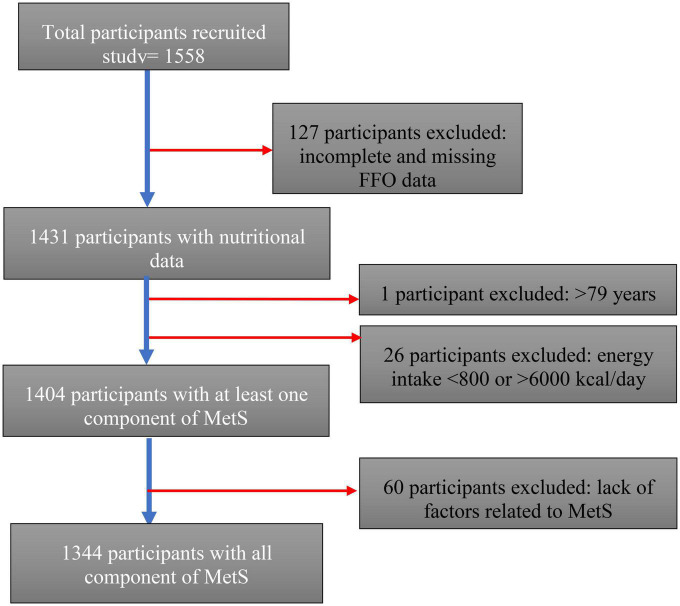
Participant sample progression.

A total of 1,558 Luxembourg residents between the ages of 25 and 81 were recruited for the ORISCAV-LUX2 study (27). Of the 1,558 participants, 127 were excluded from the analysis due to missing FFQ information or missing data, 26 due to receiving extreme values of energy, and 1 due to age over 80 years. Overall, 1,404 participants were included in our research question (descriptive analyses), with a complete dataset of nutritional characteristics and having at least one of the MetS components. When crucial data for our analysis, such as results of the five main components of MetS, were missing, it was considered an incomplete metabolic profile; thus, these cases (60 participants) were excluded. Therefore, participants with incomplete metabolic profiles were excluded from correlation and regression analyses, and 1,344 cases were considered for such analysis, as represented in the flowchart in Figure 1.

### Data collection

Participation in ORISCAV-LUX 2 research entailed three primary steps: completing a self-reported questionnaire (including socio-demographic variables), clinical and anthropometric measures by trained nurses per the standardized operating procedures, and collecting blood, urine, and hair samples in a commercial accredited Laboratory (Ketterthill, Luxembourg).

The data were obtained from questionnaires related to sociodemographic and anthropometric aspects, lifestyle, and self-reported health conditions, including a validated food frequency questionnaire (FFQ) with 174 items (29). The participants, with the assistance of a trained nurse, indicated the frequency and portion size of all consumed food items and beverages on a scale ranging from “never or rarely,” “two or more times/day,” “once a day,” “3–5 times/week,” “1–2 times/week,” and “1–3 times/month” over the previous year. The macro- and micronutrient intake was calculated by multiplying each food item’s consumption frequency by the specific nutrient content of each portion. A photo album covering portion size images of all the consumed food and beverage items was used to accurately identify the portion sizes and determine the amount of intake. The macronutrient and micronutrient intakes were calculated by translating foods and beverages into nutrients using the French Ciqual food database, which lists nutritional information for over 3,100 food items (30). Furthermore, The MDS score and its quartiles were calculated based on the FFQ and Ciqual database; the same was done regarding the AHEI. Demographics, socioeconomic factors, and details about the anthropometric and clinical measurement are all explained in more detail previously, similar to the dietary assessment (27).

### Calculation of MetS scores

The MetS as a categorical variable was defined based on the NCEP ATP III definition (Supplementary Table 1). Accordingly, the participant was considered to have MetS if he/she had three or more of the following main components: dyslipidemia, elevated arterial BP, dysregulated glucose homeostasis, abdominal obesity, and/or insulin resistance, according to the NCEP ATP III criteria (3).

However, as there is no validated tool to calculate the MetS score as a continuous variable, previous research reports have employed several methods to quantify it. We used three different standard methods in our approach to quantify the MetS.

First, we calculated the MetS score based on the weighting of its component by using the exploratory factor analysis (EFA). The scoring MetS based on EFA (MetSEFA) was calculated by subtracting the total mean of each component of MetS from values for each person and then dividing it by the total SD multiplied by the EFA coefficient for each component (31). MetSEFA = [(total mean of FBG-individuals FBG/total SD) * EFA coefficient for FBG] + [(total mean of triglyceride (TG)-individuals TG/total SD) * EFA coefficient for TG] + [(total mean of waist circumference (WC)-individuals WC/total SD) * EFA coefficient for WC] + [(total mean of SBP-individuals SBP/total SD) * EFA coefficient for SBP] + [(total mean of HDL-individuals HDL/total SD) * EFA coefficient for HDL], with FBG being fasting blood glucose, SD being standard deviation, and SBP being systolic blood pressure.

The second method (siMS score) was based on a previously published method (32), with the following equation: (2 * WC/height) + (FBG/5.6) + (TG/1.7) + (SBP/130) − (HDL/1.28 or 1.02 for women or men) with values for TG, HDL, and FBG in mmol/L, height and WC in cm.

The third quantifying method was calculated based on regression modules (MetSR) using logistic regression, and the final equation was: −19.94 + (WC * 0.072) + (FBG * 0.052) + (TG * 0.008) + (HDL * − 0.037) + (SBP * 0.025) + (gender * 1.188 or −1.188 for men or women, respectively) + (age * 0.009 or * 0.067 for men or women, respectively), with values for HDL, TG, and FBG in mg/dL.

### Calculation of Mediterranean diet score and alternative healthy eating index

#### Mediterranean diet score

The MDS was chosen in this study to assess the association of the MD diet with MetS. To calculate the MDS for this study, the intake of required food groups was obtained from the completed FFQ data. Then, the median intake of the entire population investigated was found for each food group, and after that, each participant’s food consumption was compared with the median. The MDS ranged from zero (low adherence to MD) to nine (high adherence to MD) and was calculated by combining all component ratings (33). In the end, MDS quartiles were calculated.

#### Alternative healthy eating index

The AHEI was used in this study to assess the association between a healthy diet and MetS. The AHEI uses nine nutritional components to assess food quality and may be used to offer dietary advice for healthy eating (Supplementary Table 2). Moreover, it is considered more detailed and specific compared to its predecessor, the healthy eating index (HEI), because it includes more items than the HEI, such as protein sources, red-to-white meat ratio, polyunsaturated to saturated fat, and fibers, rather than the broader categories, such as grains and all meats combined as used in the HEI. In addition, attention to the duration of multivitamin usage is also one element that distinguishes the AHEI from the original HEI and other indices (17), though we could not include it here. To construct the AHEI score for this study, relevant food items on the FFQ were allocated to their relevant food categories of each completed FFQ. The nutritional database CIQUAL was used to compute the ratio of PUFA to SFA and cereal fiber intake. The trans-fatty acid information was not available. Therefore, it was not considered in the AHEI scoring, and the AHEI was calculated for this study with only eight components. The amount of each food group was calculated in grams, and grading was done by using the highest-scoring point as a cut-off point. Each component is worth a minimum of zero and a maximum of 10 points, except for the multivitamin component (5 points is the maximum). A total AHEI score, ranging from zero (worst/unhealthy) to 75 (best/healthy), was calculated by combining all component ratings (Supplementary Table 2). In the end, the four AHEI quartiles were calculated.

## Statistical analyses

### Descriptive and correlations analyses

Statistical analyses were performed using SPSS (IBM, Chicago, IL, USA) v. 25. The normality of data distribution and equality of variance was tested by both Q-Q normality plots and the Kolmogorov–Smirnov test. Log-transformation was undertaken to obtain normal distribution when original data did not meet the normality criteria. These values were reported as median and interquartile ranges (IR), and the categorical variables were reported as numbers and percentages. For continuous data, *t*-tests were utilized (log-transformation was used for all the continuous variables), while the chi-square test was employed for categorical variables. Distributions of demographics, anthropometric and socioeconomic characteristics of participants based on gender were reported, also based on the MDS and its quartiles and the AHEI and its quartiles. In addition, the distribution of the MDS, the AHEI, and the selected nutrients of participants were reported based on gender and metabolic syndrome status. The *p*-values below 0.05 (2-tailed) were considered statistically significant.

For correlations, as our data were not normally distributed, the non-parametric correlation was performed using Spearman correlations (bivariate correlations) to assess the correlation of the dietary indices MDS and AHEI with cardiometabolic biomarkers.

*Linear regression models* were performed to assess the association of each component of MetS with MDS and AHEI. The dietary indices’ scores and the quartiles were analyzed in models A, B, and C.

Model A: the dependent variable was one of the components, and the independent variable was one of the indices, either as the continuous score or as quartiles.

Model B: Model A + adjusted for age groups and gender as confounding factors.

Model C: Model B + additionally adjusted for all sociodemographic variables and selected anthropometric variables (education, job, income, marital status, country of birth, physical activity, currently smoking, and total energy intake).

*Logistic regression models* were also performed to analyze the association of MetS (as a categorical variable) with the MDS and AHEI with three models (all dependent variables were entered in the regression models log-transformed):

Model A: the dependent variable was MetS as a categorical variable, and the independent variable is one of the indices (AHEI score, AHEI quartiles, MDS score, MDS quartiles).

Model B: Model A + adjusted for age groups and gender as confounding factors.

Model C: Model B + additionally adjusted for all sociodemographic variables and selected anthropometric variables (education, job, income, marital status, country of birth, physical activity, currently smoking, and total energy intake).

In addition, *linear regression models* were performed to assess MetS scores’ (as a continuous variable) association with MDS and AHEI. The score and the dietary indices’ quartiles were analyzed in three models A, B, and C, as the following (all dependent variables were entered in the regression models log-transformed):

Model A: the dependent variable was one of MetS scores (MetSEFA, siMS score, and MetSR), and the independent variable was one of the indices (MDS and AHEI), either as the continuous score or as quartiles.

Model B: Model A + adjusted for age groups and gender as confounding factors.

Model C: Model B + additionally adjusted for all sociodemographic variables and selected anthropometric variables (education, job, income, marital status, country of birth, physical activity, currently smoking, and total energy intake).

## Results

### Descriptive analyses

Demographic and socioeconomic distribution analyses are given in Table 1. There were significant differences between participants with MetS and participants without MetS regarding age, BMI, education, job, income, and marital status (Table 1).

**TABLE 1:** Distribution of demographics, anthropometric and socioeconomic characteristics of participants based on gender and MetS status, presented as frequency, percentage, and *P*-values, comparing women vs. men and participants without MetS vs. participants with MetS.

Variable	Total (*n* = 1,404)^e^		Women (*n* = 750)		Men (*n* = 652)		*P*-value^†^	Total (*n* = 1,344)^f^		Participant without MetS (*n* = 977)		Participant with MetS (*n* = 367)		*P*-value^†^
							
	n	%	n	%	n	%		n	%	n	%	n	%	
**Age group (years)**														
25–34.99	162	11.5	85	11.3	77	11.8	0.954	147	10.9	142	14.5	5	1.3	**<0.001**
35–44.99	315	22.4	163	21.7	152	23.2		302	22.4	266	27.2	36	9.8	
45–54.99	381	27.1	207	27.6	174	26.6		362	26.9	278	28.4	84	22.8	
55–64.99	352	25.1	191	25.5	161	24.6		342	25.4	208	21.2	134	36.5	
65–79	194	13.8	104	13.9	90	13.8		191	14.2	83	8.5	108	29.4	
**BMI (kg/m^2^)**														
Normal (<24.99)	649	46.2	416	55.5	233	35.6	**<0.001**	616	45.8	559	57.2	57	15.5	**<0.001**
Overweight (25–29.99)	493	35.1	218	29.1	275	42.0		476	35.4	330	33.7	146	39.7	
Obesity (>30)	262	18.7	116	15.5	146	22.3		252	18.7	88	9.0	164	44.6	
**Education**														
No diploma*	182	13.0	105	14.0	77	11.8	0.168	171	12.7	97	9.9	74	20.1	**<0.001**
Certified**	251	17.9	135	18.0	116	17.7		238	17.7	154	15.7	84	22.8	
Diploma***	321	22.9	158	21.1	163	24.9		311	23.1	227	23.2	84	22.8	
Tertiary****	526	37.5	277	36.9	249	38.1		506	37.6	422	43.2	84	22.8	
Did not answer	124	8.8	75	10.0	49	7.5		118	8.8	77	7.8	41	11.1	
**Job**														
Employed, maternal leave	918	65.4	467	62.3	451	69.0	**<0.001**	877	65.2	720	73.4	157	42.7	**<0.001**
Unemployed^c^	153	10.9	126	16.8	27	4.1		144	10.7	96	9.8	48	13.1	
Retired, Long leave^d^	316	22.5	147	19.6	169	25.8		308	22.9	151	15.4	157	42.7	
Did not answer	17	1.2	10	1.3	7	1.1		15	1.1	10	1.1	5	0.8	
**Income (€/month)**														
Less than 750	4	0.3	1	0.1	3	0.5	**<0.001**	4	0.3	2	0.2	2	0.5	**0.006**
750–1,499	22	1.6	14	1.9	8	1.2		18	1.3	13	1.3	5	1.3	
1,500–2,249	49	3.5	31	4.1	18	2.8		45	3.3	25	2.5	20	5.4	
2,250–2,999	78	5.6	55	7.3	23	3.5		76	5.6	53	5.4	23	6.2	
30,000–4,999	335	23.9	170	22.7	165	25.2		319	23.7	225	26.1	94	25.6	
5,000–10,000	482	34.3	235	31.3	247	37.8		469	34.9	361	36.9	108	29.4	
More than 10,000	115	8.2	47	6.3	68	10.4		109	8.1	89	10.1	20	5.4	
Did not answer	319	22.7	197	26.3	122	18.7		304	22.6	209	21.4	95	25.8	
**Marital status**														
Married	1,047	74.6	526	70.2	521	79.8	**<0.001**	1,002	74.5	705	72.1	297	80.9	**0.001**
Widow	164	11.7	86	11.5	78	11.9		157	11.6	131	13.4	26	7.1	
Divorced^b^	155	11.0	102	13.6	53	8.1		148	11.1	117	11.9	31	8.4	
Single^a^	36	2.6	35	4.7	1	0.2		36	2.6	23	2.3	13	3.5	
**Country of birth**														
Luxembourg	832	59.3	443	59.1	389	59.5	0.192	794	59.0	568	58.1	226	61.5	0.328
Portugal	110	7.8	59	7.9	51	7.8		109	8.1	79	8.1	30	8.1	
Other European countries	336	23.9	170	22.7	166	25.4		323	24.0	236	24.1	87	23.7	
Non-European country	126	9.0	78	10.4	48	7.3		118	8.7	94	9.6	24	6.5	
Physical activity	Total = 903		Women = 464		Men = 439		0.830	Total = 868		Without MetS = 656		With MetS = 212		0.452
Inactive	124	13.7	62	13.4	62	14.1		219	25.2	164	25.0	55	25.9	
Moderately active	229	25.4	121	26.1	108	24.6		530	61.0	407	62.0	123	58.0	
Active	550	60.9	281	60.6	269	61.3		119	13.7	85	13.0	34	16.0	

^†^Chi-square test was performed to find the *P*-values; Significant values are given in bold.

*Pre-primary and primary education; **CATP, Certificate of Technical and Professional Aptitude; CITP, Certificate of Technical and Professional Initiation; CCM, Certificate of Manual Capability, Diploma for Completion of Secondary Technical Studies, Diploma for Completion of Secondary General Studies; ***Technician diploma, Bac + 2 (BTS), Bac + 3 (Bachelors/Degree), Diploma from a Grande Ecole, an Engineering School; ****Bac + 4 (Masters), Bac + 5, and more (3rd Cycle, DEA, DESS, MBA, Masters, Ph.D., etc.).

^a^Single, never married, and never in a registered partnership.

^b^Divorced, separated, separated but still legally married.

^c^In school, university or training, at home, unemployed, or in search of employment.

^d^Retired or in early retirement, on long-term leave.

^e^Number of participants having at least one component of MetS assessed.

^f^Number of participants having all components of MetS assessed.

Also, there were significant differences in dietary patterns between the participants who did have MetS and participants who did not have MetS, according to plant-based protein, docosapentaenoic acid (DPA), carbohydrates, sugar, salt, zinc, sodium, potassium, dairy products, fruits, red meat, nuts and soy products and starchy vegetables (Table 2).

**TABLE 2:** Distribution of AHEI, MDS, and selected nutrient intakes of participants per day based on gender and MetS status.

Variable^a^	Participant without MetS						Participant with MetS						*P*-value* T *vs.* T
	Women (*n* = 554)	IR	Men (*n* = 423)	IR	Total (*n* = 977)	IR	Women (*n* = 159)	IR	Men (*n* = 208)	IR	Total (*n* = 367)	IR	
AHEI	35.0	15.1	37.0	14.1	36.0	14.0	37.1	15.2	36.1	14.4	37.0	14.0	0.967
MDS	5.01	2.01	4.00	3.01	5.00	2.00	5.00	31.03	5.00	2.00	5.00	3.00	0.273
Total energy (kcal)	2,118	897.1	2,683	1,182	2,328	1,120	2,171	1,048	2,619	1,116	2,426	1,155	0.072
Proteins (g)	78.4	36.3	100.8	267.0	87.8	44.2	83.4	36.3	98.2	52.7	90.1	46.1	**0.003**
Lipids (g)	59.9	49.8	65.9	57.2	61.7	52.6	57.4	45.1	62.2	49.3	59.6	46.5	0.936
SFA to MUFA ratio	0.54	0.27	0.52	0.25	0.53	0.25	0.52	0.29	0.52	0.23	0.53	0.25	0.246
Total fat (g)	108.0	56.1	129.0	65.2	115.0	64.2	108.0	58.2	123.0	68.5	114.0	65.9	0.907
PUFA (g)	19.5	12.4	22.9	13.2	20.8	13.4	20.1	14.1	20.6	15.9	20.5	14.0	0.643
EPA (g)	0.18	0.23	0.26	0.25	0.20	0.23	0.19	0.22	0.20	0.21	0.20	0.21	0.333
DHA (g)	0.26	0.32	0.31	0.37	0.29	0.32	0.27	0.30	0.28	0.29	0.28	0.30	0.488
DPA (mg)	713	683	887	749	791	687	825	651	868	652	818	672	**0.039**
Fibers, total (g)	22.5	11.6	24.1	12.8	23.1	12.2	23.7	11.8	22.8	12.3	23.1	11.7	0.479
Soluble fibers (g)	4.7	2.4	4.8	2.4	4.7	2.4	5.0	2.6	4.4	2.4	4.7	2.4	0.404
Carbohydrates (g)	194	86	243	124	214	111	210	114	233	98	225	98	**0.013**
Vegetables (g)	226	187	210	161	220	176	229	197	192	157	211	171	0.191
Simple sugars (g)	94.8	55.2	106.7	61.1	98.7	60.1	107.0	58.1	106.0	54.5	107.0	56.8	**0.041**
Sugary beverages (ml)	75.5	250.0	70.7	225.0	70.7	233.0	51.4	214.0	53.5	229.0	53.5	214.0	0.307
Non-caloric beverages (ml)	1,680	896	1,700	1,073	1,696	991	1,660	872	1,579	1,148	1,633	1,046	0.458
Vitamin C (mg)	149	102	141	100	145	99.5	159	110	141	109	145	115	0.158
Vitamin D (μg)	4.76	4.44	5.73	4.96	5.13	4.69	5.15	4.29	5.30	4.76	5.17	4.68	0.344
Vitamin E (mg)	16.8	10.0	21.0	13.6	18.5	12.0	17.1	8.65	18.5	11.9	18.0	10.3	0.133
β-Carotene (μg)	5,170	4,509	4,947	3,969	5,057	4,096	5,464	4,052	4,479	3,595	4,842	3,758	0.166
Energy (kcal)	2,118	897	2,683	1,182	2,328	1,120	2,171	1,048	2,619	1,116	2,426	1,155	0.063
Water (ml)	3,002	1,074	3,122	1,365	3,072	1,196	3,041	1,279	3,118	1,392	3,080	1,342	0.826
Alcohol (ml)	3.82	7.65	9.33	15.40	5.86	11.00	2.88	7.74	9.22	16.3	5.64	12.3	0.575
Zinc (mg)	10.90	4.84	14.29	6.24	12.10	6.19	11.50	4.77	13.2	7.22	12.4	6.47	**0.009**
Selenium (μg)	86.0	35.2	103.4	43.8	92.3	40.0	85.3	44.1	95.7	48.8	92.1	45.2	0.299
Sodium (mg)	2,882	1,511	3,855	2,135	3,261	1,909	3,044	1,748	3,797	1,976	3,416	1,942	**0.012**
Potassium (mg)	3,338	1,405	3,714	1,587	3,472	1,503	3,606	1,474	3,665	1,679	3,643	1,553	**0.006**
Grains (g)	109	91	136	111	117	96	116	106	131	115	121	113	0.156
Dairy products (g)	169	199	170	177	170	186	199	193	178	220	189	207	**0.010**
Fruits (g)	286	256	275	261	281	261	335	308	304	260	316	303	**0.006**
Legumes (g)	6.6	21.4	10.0	13.3	6.67	21.4	6.67	13.3	6.6	13.3	6.6	13.3	0.664
Red meat (g)	100	93	154	105	121	112	106	70	159	137	131	124	**0.004**
Nuts and soya (g)	1.67	10.70	0.99	10.70	1.67	10.70	0.99	10.70	0.99	6.67	0.99	10.0	**0.039**
Fish (g)	64.6	65.3	78.14	72.0	70.8	68.2	64.1	63.2	73.8	70.7	70.1	68.6	0.880
Polyphenols (mg)	2,511	1,659	2,575	1,620	2,539	1,656	2,626	1,332	2,484	1,743	2,504	1,601	0.463
Fast food^b^ (g)	72	86	120	10	92	100	85	88	107	122	98.2	112	0.199
Protein-rich food (g)	184	124	245	162	209	139	185	119	236	176	214	866	0.117
Starchy vegetables (g)	49.7	59.9	62.8	60.7	55.4	61.3	56.9	73.3	70.9	77.2	64.2	77.14	**<0.001**

Shown by the median and interquartile range (IR), and *P*-value compares total participants without MetS vs. participants with MetS.

*Log of the variables was used for the independent sample *t*-test.

^a^Median and interquartile ranges were reported for all the variables because these were not normally distributed.

^b^Fast foods and ready-to-eat meals.

AHEI, alternative healthy eating index; MDS, Mediterranean diet score; SFA, saturated fatty acids; PUFA, polyunsaturated fatty acids; EPA, eicosapentaenoic acid; DHA, docosahexaenoic acid; DPA, docosapentaenoic acid; IR, interquartile range; T *vs*. T, comparing total participants without MetS with total participants with MetS. *Significant values are given in bold.

In addition, a comparison of dietary intakes based on categories of MetS components are represented in Table 3. The results of the in-depth investigation showed that participants with SBP higher than the ATP III cut-off (≥130) significantly consumed more energy, red meat, grains, starchy vegetables, total fat, SFA, sodium, simple sugars, alcohol, and less fish and DHA than participants with SBP lower than the cut-off (<130). Similar findings were encountered for DBP. Regarding other components, participants with WC higher than the cut-off [≥102 (men) and ≥88 (women)] consumed significantly more red meat, starchy vegetables, dairy products, cholesterol, sodium, and alcohol comparing participants with WC lower than the cut-off [<102 (men) and <88 (women)]. Similar results were obtained comparing participants based on FBG and TG and in the reverse for HDL categories.

**TABLE 3:** Comparison (median and IR) of intake of food groups and selected nutrients based on MetS components.^a^

Variable	SBP (mmHg)			DBP (mmHg)			WC (cm)			HDL (mg/dL)			FBG (mg/dL)			Triglycerides (mg/dL)		
	≥130	<130	*P*-value	≥85	<85	*P*-value	≥102 (men) ≥88 (women)	<102 (men) <88 (women)	*P*-value	<40 (men) <50 (women)	≥40 (men) ≥50 (women)	*P*-value	≥100	<100	*P*-value	≥150	<150	*P*-value
Total energy (kcal/d)	2,507 (1,115)	2,289 (1,096)	**<0.001**	2,471 (1,137)	2,330 (1,103)	0.114	4,208 (1,143)	2,361 (1,112)	0.268	2,501 (1,116)	2,327 (1,102)	0.188	2,551 (1,367)	2,357 (1,098)	**0.029**	2,656 (1,196)	2,310 (1,097)	**<0.001**
Fish group (g/d)	66.3 (64.1)	73.1 (68.9)	**0.005**	68.1 (63.1)	71.3 (69.3)	0.222	72.7 (69.5)	70.3 (68.4)	0.704	70.2 (66.5)	73.4 (76.8)	0.559	69.9 (67.3)	84.1 (70.5)	0.178	70.9 (76.9)	70.8 (67.8)	0.857
Red meat (g/d)	131.1 (118.1)	120.2 (118.8)	**0.024**	135.6 (126.5)	120.6 (115.8)	**0.003**	138.7 (127.7)	118.7 (110.5)	**<0.001**	145.5 (123.6)	119.8 (112.9)	**0.001**	143.8 (134.7)	119.7 (113.2)	**0.003**	165.5 (125.4)	118.3 (106.1)	**<0.001**
Fruits (g/d)	296.5 (288.7)	289.8 (271.9)	0.171	283.1 (281.6)	300.1 (276.3)	0.122	309.8 (280.4)	282.2 (274.7)	0.200	292.2 (282.1)	296.5 (273.5)	0.335	310.0 (244.2)	289.5 (277.2)	0.401	285.3 (298.9)	296.1 (271.1)	0.792
Vegetables (g/d)	215.1 (177.6)	217.3 (166.1)	0.507	194.8 (175.1)	221.3 (167.9)	**0.017**	217.7 (175.7)	216.7 (170.1)	0.870	213.4 (179.0)	219.3 (171.1)	0.740	226.9 (202.6)	216.4 (168.2)	0.582	220.0 (181.2)	217.6 (172.6)	0.398
Starchy veg (g/d)	67.1 (79.5)	53.5 (59.0)	**<0.001**	62.8 (76.0)	56.1 (59.0)	**0.045**	64.2 (76.3)	56.1 (57.9)	**0.013**	62.8 (75.3)	56.1 (60.7)	**0.013**	64.2 (66.9)	56.1 (62.6)	**0.002**	62.8 (72.5)	56.1 (62.6)	**0.010**
Fast foods^b^ (g/d)	96.6 (109.1)	92.2 (98.9)	0.096	95.6 (117.2)	93.9 (99.1)	0.758	99.9 (106.3)	92.0 (101.1)	0.385	110.0 (116.8)	87.4 (100.3)	**0.001**	102.5 (116.6)	92.0 (99.0)	0.352	121.2 (134.5)	90.1 (95.8)	**<0.001**
Grains (g/d)	126.2 (101.2)	115.9 (100.4)	**0.012**	120.2 (111.9)	119.2 (98.7)	0.710	115.8 (106.3)	120.7 (99.1)	0.414	132.3 (117.2)	117.1 (97.1)	**0.041**	134.0 (113.2)	117.5 (98.9)	0.072	129.2 (89.9)	117.6 (102.8)	0.146
Dairy (g/d)	179.7 (205.7)	175.6 (199.0)	0.178	186.3 (198.0)	175.1 (199.8)	0.296	189.7 (215.0)	171.4 (186.5)	**0.009**	182.5 (215.0)	174.5 (194.6)	0.932	180.1 (208.6)	175.0 (196.0)	0.605	184.5 (199.9)	174.5 (198.5)	0.338
Total fiber (g/d)	23.6 (12.5)	22.9 (11.8)	0.051	22.6 (12.2)	23.5 (12.1)	0.281	23.2 (12.3)	23.2 (12.1)	0.987	23.6 (12.5)	23.0 (11.9)	0.518	24.0 (12.8)	23.0 (11.9)	0.235	22.8 (11.9)	23.2 (12.2)	0.894
Soluble fiber (g/d)	4.8 (2.5)	4.7 (2.4)	0.054	4.5 (2.6)	4.8 (2.4)	0.198	4.7 (2.6)	4.7 (2.4)	0.456	4.8 (2.4)	4.7 (2.4)	0.449	4.8 (2.7)	4.7 (2.4)	0.199	4.5 (2.5)	4.8 (2.4)	0.807
Total fat (g/d)	119.9 (64.1)	114.6 (63.4)	**0.025**	119.7 (66.1)	115.0 (63.5)	0.243	118.2 (63.6)	115.6 (65.4)	0.492	121.0 (66.3)	115.3 (64.3)	0.464	119.6 (74.1)	115.6 (63.8)	0.454	124.6 (64.9)	114.5 (64.7)	**0.003**
EPA (g/d)	0.18 (0.21)	0.20 (0.23)	0.158	0.18 (0.21)	0.20 (0.23)	0.584	0.19 (0.22)	0.20 (0.23)	**0.045**	0.19 (0.23)	0.20 (0.23)	0.878	0.20 (0.27)	0.19 (0.23)	0.069	0.20 (0.22)	0.19 (0.23)	0.493
DHA (g/d)	0.25 (0.29)	0.30 (0.32)	**0.037**	0.26 (0.29)	0.29 (0.32)	0.248	0.30 (0.31)	0.27 (0.32)	0.093	0.28 (0.32)	0.28 (0.31)	0.895	0.29 (0.40)	0.28 (0.31)	0.106	0.28 (0.33)	0.28 (0.31)	0.618
MUFA (g/d)	48.9 (25.1)	46.1 (26.3)	0.074	48.2 (26.7)	46.4 (26.3)	0.464	47.3 (25.3)	46.5 (26.8)	0.399	48.5 (27.0)	46.7 (26.5)	0.349	49.0 (32.7)	46.5 (26.0)	0.249	50.6 (27.1)	46.3 (26.3)	**0.004**
PUFA (g/d)	21.5 (13.5)	20.3 (13.5)	0.270	21.4 (13.4)	20.5 (13.4)	0.851	20.7 (13.3)	20.8 (13.5)	0.782	20.8 (13.8)	20.8 (13.3)	0.938	21.0 (16.8)	20.8 (13.5)	0.893	21.4 (14.6)	20.7 (13.6)	0.114
SFA (g/d)	41.8 (24.5)	38.8 (21.8)	**0.001**	42.4 (24.8)	39.1 (21.8)	**0.030**	40.0 (23.4)	39.4 (22.4)	0.285	40.9 (24.6)	39.3 (22.4)	0.960	40.2 (23.9)	39.4 (22.3)	0.495	44.4 (21.8)	38.8 (22.3)	**<0.001**
Cholesterol (mg/d)	372.8 (214.9)	349.8 (197.1)	0.058	367.9 (220.8)	353.5 (198.8)	0.126	371.5 (215.3)	348.1 (206.5)	**<0.001**	381.8 (199.2)	349.8 (201.4)	**0.035**	376.7 (342.2)	351.1 (196.2)	**0.005**	402.9 (230.0)	344.7 (192.8)	**<0.001**
Phenolics (mg/d)	2,678 (1,474)	2,480 (1,707)	0.190	2,691 (1,667)	2,506 (1,642)	0.877	2,562 (1,754)	2,538 (1,617)	0.380	2,382 (1,627)	2,575 (1,616)	0.098	2,584 (1,465)	2,522 (1,659)	0.088	2,448 (1,790)	2,553 (1,632)	0.486
Beta-carotene (μg/d)	5,071 (4,451)	4,966 (4,004)	0.609	4,854 (4,111)	5,042 (4,027)	0.169	4,977 (3,990)	4,994 (4,143)	0.951	4,697 (4,122)	5,047 (3,959)	0.781	5,045 (3,977)	4,992 (4,095)	0.982	4,695 (3,774)	5,044 (4,112)	0.590
Sodium (mg/d)	3,512 (2,037)	3,193 (1,821)	**<0.001**	3,526 (2,156)	3,236 (1,830)	**0.005**	3,460 (1,940)	3,229 (1,936)	**0.003**	3,581 (1,840)	3,239 (1,918)	**0.001**	3,642 (2,248)	3,267 (1,891)	**0.008**	3,911 (2,201)	3,225 (1,842)	**<0.001**
Simple sugars (g/d)	107.6 (60.2)	98.7 (59.6)	**0.001**	101.6 (56.0)	100.6 (60.4)	0.602	101.6 (62.1)	100.5 (59.2)	0.717	98.5 (59.0)	101.4 (58.7)	0.813	99.8 (68.2)	100.9 (57.7)	0.603	107.7 (60.34)	99.7 (58.3)	**0.024**
Alcohol (g/d)	7.7 (13.8)	5.1 (10.1)	**0.001**	7.5 (14.7)	5.27 (11.1)	**0.001**	4.6 (11.1)	6.4 (12.0)	**0.028**	4.4 (8.2)	6.4 (12.2)	**0.004**	9.0 (16.8)	5.5 (11.0)	**0.002**	8.9 (17.1)	5.4 (10.9)	**<0.001**

Significant values are given in bold.

^a^All *P*-values are reported based on log-transformed data using the *t*-test.

^b^Fast foods and ready-to-eat meals.

IR, interquartile range; SBP, systolic blood pressure; DBP, diastolic blood pressure; WC, waist circumference; HDL, high-density lipoprotein cholesterol; FBG, fasting blood glucose levels; TG, triglycerides; Veg, vegetables; EPA, eicosapentaenoic acid; DHA, docosahexaenoic acid; MUFA, monounsaturated fatty acids; PUFA, polyunsaturated fatty acids; SFA, saturated fatty acids; Phenolics, total phenolics.

### Correlation analyses

The correlation analyses between MDS and cardiometabolic markers revealed statistically significant inverse correlations with BMI, waist circumference (WC), TG, SBP, and DBP. Furthermore, a moderate positive correlation was observed between the MDS and the AHEI. The AHEI showed no statistically significant correlation with cardiometabolic markers (Table 4); however, significant results were observed in regression models adjusted for confounding factors.

**TABLE 4:** Correlation of dietary indices MDS and AHEI with cardiometabolic biomarkers.

Variables	MDS		AHEI	
	CC	*P*-value*	CC	*P*-value*
BMI (kg/m^2^)	−0.118	<**0.001**	−0.043	0.115
Insulin (μg/L)	−0.031	0.252	0.024	0.373
HbA1c (%)	0.020	0.462	0.046	0.093
HOMA-IR	−0.040	0.143	0.020	0.471
TG (mg/dL)	−0.068	**0.013**	−0.019	0.496
Total cholesterol (mg/dL)	−0.012	0.667	−0.014	0.614
LDL (mg/dL)	0.001	0.975	0.018	0.500
MDS			0.473	<**0.001**
AHEI	0.473	<**0.001**		

Presented as correlation coefficient (CC) and *P*-value.

*Spearman test, non-parametric correlation.

Significant values are given in bold.

Correlation is significant at the 0.05 level (2-tailed).

AHEI, alternative healthy eating index; MDS, Mediterranean diet score; BMI, body mass index; WC, waist circumference; FBG, fasting blood glucose levels; HbA1c, glycated hemoglobin; HOMA, homeostatic model assessment of insulin resistance; TG, triglycerides; HDL, high-density lipoprotein cholesterol; LDL, low-density lipoprotein cholesterol.

### Regression models

Linear regression analyses of MDS and AHEI with MetS components in final models revealed statistically significant negative associations between MDS and BMI, WC, SBP, and DBP (Table 5, model C). Similar findings were observed for MDS when investigated by quartiles. Regarding the AHEI score, negative associations were observed with the BMI, WC, fasting blood glucose (FBG), SBP, and DBP. For the AHEI quartiles, negative associations were found with BMI, WC, and SBP only (Supplementary Table 3).

**TABLE 5:** Association of the dietary indices MDS and AHEI with MetS components, as derived by linear regression (beta non-standardized, 95% CI, *P*-value**, and strength of linear association R).

Dependent^a^/ Independent variable	Model-A					Model-B					Model-C Full				
	Beta	95% CI		*P*-value	*R*	Beta	95% CI		*P*-value	*R*	Beta	95% CI		*P*-value	*R*
BMI*/MDS	−0.0054	−0.0080	−0.0027	<**0.001**	0.106	−0.0050	−0.0075	−0.0024	<**0.001**	0.308	−0.0067	−0.0099	−0.0036	<**0.001**	0.375
WC/MDS	−0.0048	−0.0071	−0.0026	<**0.001**	0.113	−0.0039	−0.0059	−0.0020	<**0.001**	0.517	−0.0048	−0.0072	−0.0024	<**0.001**	0.559
FBG/MD	−0.0017	−0.0039	0.0005	0.136	0.041	−0.0015	−0.0036	0.0005	0.138	0.384	−0.0004	−0.0027	0.0017	0.673	0.427
TG/MD	−0.0095	−0.0170	−0.0020	**0.013**	0.068	−0.0071	−0.0142	−0.00006	**0.048**	0.335	−0.0026	−0.0115	0.0063	0.565	0.396
HDL/MDS	0.0017	−0.0021	0.0056	0.376	0.024	−0.0009	−0.0043	0.0025	0.596	0.476	−0.0001	−0.0043	0.0041	0.957	0.536
SBP/MDS	−0.0034	−0.0054	−0.0013	**0.001**	0.087	−0.0030	−0.0048	−0.0012	**0.001**	0.510	−0.0038	−0.0060	−0.0016	**0.001**	0.530
DBP/MDS	−0.0036	−0.0058	−0.0015	**0.001**	0.091	−0.0033	−0.0053	−0.0013	**0.001**	0.335	−0.0034	−0.0060	−0.0009	**0.008**	0.344
BMI*/AHEI	−0.0001	−0.0005	0.0002	0.460	0.020	0.0001	−0.001	0.0005	0.139	0.294	−0.0001	−0.002	−0.0002	**0.001**	0.263
WC/AHEI	0.0001	−0.0001	0.0005	0.351	0.025	−0.00003	−0.0003	0.0002	0.809	0.508	−0.0007	−0.0011	−0.0002	**0.002**	0.559
FBG/AHEI	−0.00008	−0.0004	0.0002	0.631	0.013	0.0002	−0.0005	0.00008	0.143	0.384	−0.0005	−0.0009	−0.00009	**0.017**	0.436
TG/AHEI	−0.0006	−0.0017	0.0005	0.297	0.028	−0.0010	−0.0020	0.00006	0.064	0.334	−0.0008	−0.0025	0.0008	0.324	0.359
HDL/AHEI	−0.0004	−0.0010	0.0001	0.123	0.042	−0.0002	−0.0007	0.0002	0.333	0.477	−0.00003	−0.0008	0.0007	0.933	0.506
SBP/AHEI	−0.00007	−0.0003	0.0002	0.634	0.013	−0.0002	−0.0005	0.000007	0.056	0.506	−0.0006	−0.0010	−0.0002	**0.004**	0.533
DBP/AHEI	−0.0003	−0.0006	−0.00009	**0.044**	0.054	−0.0004	−0.0007	−0.0001	**0.003**	0.333	−0.0005	−0.0010	−0.00009	**0.021**	0.345

^a^All dependent variables were entered in the models as log-transformed.

Model A: the dependent variable was one of the components, and the independent variable was one of the indices as the continuous score.

Model B: the dependent variable was one of the components, and the independent variables were one of the indices as the continuous score, in addition to age groups and gender as confounding factors.

Model C: the dependent variable was one of the components, and the independent variable was one of the indices as the continuous score, and the confounding factors were age group, gender, and all sociodemographic variables and selected anthropometric variables (education, job, income, marital status, and country of birth, physical activity, currently smoking, and total energy intake).

AHEI, alternative healthy eating index; MDS, Mediterranean diet score; BMI, body mass index; WC, waist circumference; FBG, fasting blood glucose levels; TG, triglycerides; HDL, high-density lipoprotein cholesterol; SBP, systolic blood pressure; DBP, diastolic blood pressure.

*The BMI is not a component of the MetS and is analyzed for additional information.

**Significant values are given in bold.

The final model (C) of logistic regression analysis revealed no statistically significant association between MetS and the dietary indices MDS and AHEI, neither when presented as scores nor as quartiles (Supplementary Table 4).

Linear regression analyses of MetS scores (based on the EFA scoring model; MetSEFA) with MDS (as a score and as quartiles) in the three models revealed statistically significant negative associations; similar findings were observed regarding MetS scores (based on the regression scoring model; MetSR) and MDS. Moreover, linear regression analyses of the MetS score presented as the siMS score with the MDS revealed statistically significant negative associations only in model A (Table 6).

**TABLE 6:** Association of MetS scores with MDS and AHEI, as calculated by linear regression (beta non-standardized, 95% CI, *P*-value*, and strength of linear association R).

Models	Dependent^a^/ Independent variable	Beta	*P*-value	95%CI		*R*
Model A	siMS/MDS	−0.062	**0.023**	−0.211	−0.016	0.062
	siMS/AHEI	−0.025	0.366	−0.021	0.008	0.025
	EFA/MDS	−0.125	<**0.001**	−0.365	−0.148	0.125
	EFA/AHEI	−0.047	0.085	−0.031	0.002	0.047
	MetSR/MDS	−0.086	**0.002**	−0.309	−0.073	0.086
	MetSR/AHEI	0.031	0.258	−0.008	0.028	0.031
Model B	siMS/MDS	−0.049	0.061	−0.184	0.004	0.277
	siMS/AHEI	−0.041	0.115	−0.026	0.003	0.276
	EFA/MDS	−0.122	<**0.001**	−0.344	−0.156	0.518
	EFA/AHEI	−0.079	<**0.001**	−0.038	−0.010	0.510
	MetSR/MDS	−0.054	<**0.001**	−0.189	−0.051	0.814
	MetSR/AHEI	−0.020	0.205	−0.017	0.004	0.812
Model C	siMS/MDS	−0.028	0.370	−0.176	0.066	0.384
	siMS/AHEI	−0.054	0.152	−0.037	0.006	0.385
	EFA/MDS	−0.118	<**0.001**	−0.346	−0.120	0.534
	EFA/AHEI	−0.133	<**0.001**	−0.059	−0.019	0.533
	MetSR/MDS	−0.053	**0.004**	−0.197	−0.039	0.848
	MetSR/AHEI	−0.056	**0.010**	−0.033	−0.004	0.848

^a^All dependent variables were entered in the models as log-transformed.

Model A: the dependent variable was one of the MetS Score, and the independent variable was one of the indices, as the continuous score.

Model B: the dependent variable was one of the MetS Score, and the independent variables were one of the indices as the continuous score, in addition to age groups and gender as confounding factors.

Model C: the dependent variable was one of the MetS Score, and the independent variable was one of the indices as the continuous score, and the confounding factors were age group, gender, and all sociodemographic variables and selected anthropometric variables (education, job, income, marital status, and country of birth, physical activity, currently smoking, and total energy intake).

*Significant values are given in bold.

siMS: MetS score; EFA: Exploratory factor analysis; MetSR: MetS based on regression models.

The linear regression analyses between the MetSEFA score and the AHEI (both the score and the quartiles) in model B and C show a significant negative association. Regarding the MetSR, there was a significant negative association with the AHEI in model C. However, there was no significant association between AHEI and MetS score as a siMS score with the AHEI (Table 6; Supplementary Table 5).

## Discussion

In this nationwide cross-sectional study, we investigated the association between two prominent dietary indices, the MDS and AHEI, with MetS (both as a categorical and continuous outcome) and its components. Logistic regression models did not reveal significant associations between the MDS or AHEI and the odds of MetS (as a categorical variable). However, when employing MetS as a continuous variable, inverse associations between MDS and AHEI and MetS were found partly in the fully adjusted models. Furthermore, based on the final adjusted linear regression models, the MetS components BMI, WC, SBP, and DBP were significantly inversely associated with the MDS, and BMI, WC, SBP, DBP, and FBG with the AHEI.

The inverse associations of the MDS with BMI and WC are well-aligned with the findings from previous studies and with meta-analyses (3, 34). As noted earlier, MD has been reported to positively affect central adiposity in persons with MetS (3, 34). The MD’s high intake of fruits, vegetables, fibers, whole grains, and unsaturated fatty acids (MUFA and PUFA) characteristics has been reported to decrease adiposity, leading to improvements in BMI and, thus also, WC. The mechanistic aspects involved may include antioxidant, anti-inflammatory, and also prebiotic-like effects (35), all of which having metabolic advantages on the cardiovascular system. The influence of the MD on the human gut microbiota has been met recently with increased interest, suggesting that the gut microbiota of people following a Mediterranean-type diet was significantly different in terms of diversity and richness (e.g., *F. prausnitzii* and *B. cellulosilyticus*) from people on a Western-type diet (36). Suggested possible mechanisms included bacterial metabolic effects in the colon, including plant-derived polysaccharide degradation, SCFA production out of dietary fiber, and increased excretion of secondary bile acids (36). However, our present knowledge of the cause-effect associations between gut microbiota and the risk of diseases is incomplete and requires further controlled intervention trials to understand the causal relationship between gut microbiota and dietary patterns, including the MD. In addition, direct effects *via* reduced energy intake are also plausible. For example, due to their high protein content, the nuts included in the MD may positively affect satiety, leading to a decreased caloric intake and, thus, a reduction in BMI (37). In the present study, participants with MetS vs. without MetS–in addition to consuming fewer nuts–also consumed more salt, red meat, and simple sugars (Table 2), which are all characteristics of a more Western-type diet but are typically low in the MD (14), and which could negatively affect MetS (8, 14). Especially simple sugars, due to their pro-inflammatory aspects and impacting insulin sensitivity (38, 39), have been related to the risk of MetS (40), and red meat, due to its high heme-iron content and less favorable fatty acid profile, have also been related to MetS (41). However, it was also observed that persons with MetS consumed more fruits and starchy vegetables (mostly potatoes) than persons without MetS, but of course, reverse causality (persons with MetS being more prudent with their dietary habits) cannot be excluded from our study.

Regarding the association of the odds of MetS and the MDS and the AHEI, the logistic regression results were surprising, as they revealed a non-significant association between MetS and the MDS or the AHEI, even in the final adjusted models. This may be due to the fact that MetS was reported as a categorical variable with a bivariate outcome (yes, no), which likely decreased statistical power compared to continuous variables such as the individual components. Accordingly, in future analyses, it would be recommendable to extend analyses and to study the effects of dietary indices on a “MetS score,” e.g., the Metabolic Syndrome Severity Score as a continuous variable that allows for better powered statistical analyses. Indeed, such MetS scores have been proposed (32, 42); however, no validated or globally accepted, or recommended single score exists. When using MetS as a continuous variable, the EFA-based score and especially the MetSR score resulted in a significant inverse association with a high regression coefficient (Table 6) between both the MDS and the AHEI and MetS, which is more in line with the results of the MetS components. Both the MDS and AHEI were similarly associated with MetS regarding the regression coefficient and beta.

Our findings regarding blood pressure (BP) measurements are in line with previous research studies and meta-analysis outcomes that also showed an inverse association of the MDS with both SBP and DBP (43, 44). The effects of MD on BP may have several reasons. For instance, the MD is rich in polyphenols such as flavonoids that can improve the formation of nitric oxide, which can enhance vasodilation and endothelial function (45). Carotenoids, another group of secondary plant metabolites, could have anti-inflammatory factors that are also important for vessel health (46). In addition to acting in an antioxidant and anti-inflammatory fashion *via* transcription factors, this can also improve vessel health (47). The intake of fruits and vegetables and less processed foods lowers the burden of sodium intake, which has been strongly associated with BP (48). As in our study, polyphenol and also carotenoid intake did not differ between persons with and without MetS; perhaps the higher salt intake in persons with MetS could have rather been playing a more important role. Though not all studies found significant effects of MD on BP, e.g., in the study of Thomopoulos et al., it was argued that even a marginal effect could contribute to improved CVD risk, as 1 mm Hg improvement in BP can lead to a 2% improvement of CVD risk (49).

Regarding blood lipids, such as triglycerides and HDL-C, our results did not reveal an inverse association in our final adjusted model. Our finding is in line with the results by Godos et al., who found a non-significant association between MDS and TG (3). Other studies, though, have shown a significant inverse association between adherence to MD and TG (34). However, a systematic review concluded that the MD significantly influences HDL-C functionality (antioxidant and anti-inflammatory properties), size, and composition. Specifically, there was an expressed need to clarify MD-derived modulations of HDL-C regarding the determination of HDL-C subgroups, some of which are rather pro- (e.g., HDL2) and some anti-inflammatory (e.g., HDL3) (50). Therefore, our study’s lack of significant results could be due to the lack of insights into relevant HDL-C subgroups, differing by size and lipid composition. In addition, it is likely that population differences and additional possible confounders, such as genetic background or environmental variables, might account for these differences in findings.

Fasting blood glucose, another hallmark component of MetS, did also not associate significantly with MD. This outcome is, on one side, similar to the findings of previous meta-analyses (3, 34). On the other side, Shah et al., in their meta-analysis, described the MD as the best anti-diabetic diet, as it significantly decreased the risk of T2D (51), which has been emphasized in the general literature, as reviewed previously (20). It is possible that FBG exhibited too high variability in the present study, and other markers, such as HbA1C, would have been a more suitable predictor of blood glucose status. FBG is also strongly influenced by physical activity (51), as are blood lipids; however, as we took it into account as a possible confounder, we do not think this perturbed our analysis.

Regarding the AHEI, our investigations found significant inverse associations between the AHEI and BMI, WC, FBG, and BP. These results are largely in accordance with previous findings from meta-analyses and research studies (52–56). Such results are not surprising because increasing the intake of healthy food groups, including fruits, vegetables, and unsaturated fat, and avoiding unhealthy food groups, such as red meat and saturated fat, can supply the body with vitamins and secondary plant metabolites such as polyphenols (e.g., flavonoids) and carotenoids that can improve vascular function, insulin sensitivity, thrombosis, and inflammation (57).

Comparing the association of the MDS and the AHEI with MetS components, it was found that the MDS had a similar association with all MetS components than the AHEI. For instance, improving both scores by one-third, i.e., 3 units in MDS score would reduce WC by 1.43%, and an increase in 25 units of AHEI would reduce WC by 1.74%. However, there are some differences in the scoring algorithm between MDS and the AHEI, such as the fish group that only exists in the MDS. Fish is rich in anti-inflammatory omega-3 fatty acids, which can improve the MetS and its components (58), while the AHEI scoring algorithm reflects the fatty acid as a ratio of MUFA to SFA. In this case, MDS may be more capable of capturing the benefit of fatty acids regarding the MetS thanks to its scoring algorithm that includes the fish group. Although fish intake did not differ between persons with MetS and those without, this differs when focusing on components of MetS. For example, participants with SBP higher than the ATP III cut-off (≥130) significantly consumed less fish and DHA than participants with SBP lower than the ATP III cut-off (<130) (Table 3). However, the intake of some food groups, such as fruits and vegetables, and in line with these, the intake of beta-carotene and polyphenols between people with and without MetS, as well as its components, were not significantly different.

The present study has limitations. First, it was a cross-sectional study; thus, no causality can be inferred from such epidemiological studies. This may be overcome in the future by prospective cohort studies or even randomized controlled trials. Second, our data was not fully representative of the Luxembourg population regarding age, BMI, and place of residence. Accordingly, the results cannot be generalized to the general population. This may be amended by future larger trials with superior stratification designs.

Moreover, Luxembourg’s geographical location may influence the MD’s operational definition and whether it can accurately reflect the typical Mediterranean dietary pattern in its original location. Therefore, there may be a need for an adapted MDS for non-Mediterranean countries (59). Last but not least, diet scores that adapt for energy as part of the scoring algorithm, namely “the relative MDS,” may provide a stronger insight into how MD diet composition is related to body weight, which is—*via* WC—a core component of MetS. Still, we have selected to assess the more commonly used unadjusted scores. The same applies to the AHEI.

Strengths of the study include that a trained nurse collected the FFQ, which was not a self-ported questionnaire; thus, measurement bias was reduced. Moreover, using the FFQ as a tool for reporting dietary intake is more appropriate and precise than some other techniques, such as food records. The FFQ also contained a large number of food items (174 items), and it was matched with a local food composition database (Ciqual from France) to estimate nutrient intake. Finally, we had anthropometric measurements rather than relying on self-reported measures often used in similar large data sets, and a commercially accredited laboratory in Luxembourg carried out the biological measurements.

## Conclusion

According to our knowledge, this is the first study to observe an association of the MDS and the AHEI with MetS and its components across Luxembourgish residents. Our study found significant inverse associations between MDS and BMI, WC, and blood pressure as components of MetS and, similarly, inverse associations between the AHEI and MetS components (BMI, WC, FBG, and blood pressure). It was also shown that a MetS severity score that is continuous was more aligned with findings from the components than the classical categorical definition. Accordingly, results support that adherence to the MD or healthy eating patterns can improve cardiovascular disease risk factors, including MetS. Further research is needed to examine the association of MDS and AHEI in more detail and focus on additional biomarkers of MetS components, such as markers of inflammation and/or oxidative stress. Finally, to validate that the MD and the AHEI might be taken as a first option for the primary prevention of MetS, more research on high-risk patients is needed, preferably within the frame of intervention studies.

## Data availability statement

The original contributions presented in this study are included in this article/Supplementary material, further inquiries can be directed to the corresponding author.

## Ethics statement

The studies involving human participants were reviewed and approved by CNER–Comité National d’Ethique de Recherche. The patients/participants provided their written informed consent to participate in this study.

## Author contributions

FV and KA performed the statistical analyses, interpreted the data, and drafted the manuscript. FV and TB provided expertise and oversight on the intellectual content. TB was involved in critically reviewing the manuscript. All authors read and agreed to the published version of the manuscript.

## References

[1] Saklayen M. The global epidemic of the metabolic syndrome. *Curr Hypert Rep.* (2018) 20:1–8. 10.1007/s11906-018-0812-z 29480368 PMC5866840

[2] Alkerwi A Donneau A Sauvageot N Lair M Scheen A Albert A Prevalence of the metabolic syndrome in Luxembourg according to the Joint Interim Statement definition estimated from the ORISCAV-LUX study. *BMC Publ Health.* (2011) 11:4. 10.1186/1471-2458-11-4 21205296 PMC3024931

[3] Godos J Zappalà G Bernardini S Giambini I Bes-Rastrollo M Martinez-Gonzalez M. Adherence to the Mediterranean diet is inversely associated with metabolic syndrome occurrence:a meta-analysis of observational studies. *Int J Food Sci Nutr.* (2017) 68:138–48. 10.1080/09637486.2016.1221900 27557591

[4] Detection NCEPEPo, Adults ToHBCi. Third report of the national cholesterol education program (NCEP) expert panel on detection, evaluation, and treatment of high blood cholesterol in adults (adult treatment panel III): the program. *Circulation.* (2002) 106:3143–421. 10.1161/circ.106.25.3143 12485966

[5] Alberti K Zimmet P Shaw J. The metabolic syndrome—a new worldwide definition. *Lancet.* (2005) 366:1059–62. 10.1016/S0140-6736(05)67402-8 16182882

[6] Mottillo S Filion K Genest J Joseph L Pilote L Poirier P The metabolic syndrome and cardiovascular risk:a systematic review and meta-analysis. *J Am Coll Cardiol.* (2010) 56:1113–32. 10.1016/j.jacc.2010.05.034 20863953

[7] Bianchi C Penno G Romero F Del Prato S Miccoli R. Treating the metabolic syndrome. *Exp Rev Cardiov Ther.* (2007) 5:491–506. 10.1586/14779072.5.3.491 17489673

[8] Fabiani R Naldini G Chiavarini M. Dietary patterns and metabolic syndrome in adult subjects:a systematic review and meta-analysis. *Nutrients.* (2019) 11:2056. 10.3390/nu11092056 31480732 PMC6770202

[9] Chartrand D Murphy-Després A Alméras N Lemieux I Larose E Després J. Overweight, obesity, and CVD risk:a Focus on visceral/ectopic fat. *Curr Atheroscl Rep.* (2022) 24:185–95. 10.1007/s11883-022-00996-x 35235165

[10] Després J Lemieux I. Abdominal obesity and metabolic syndrome. *Nature.* (2006) 444:881–7. 10.1038/nature05488 17167477

[11] van Namen M Prendergast L Peiris C. Supervised lifestyle intervention for people with metabolic syndrome improves outcomes and reduces individual risk factors of metabolic syndrome:a systematic review and meta-analysis. *Metabolism.* (2019) 101:153988. 10.1016/j.metabol.2019.153988 31672441

[12] Rodríguez-Monforte M Flores-Mateo G Sánchez E. Dietary patterns and CVD:a systematic review and meta-analysis of observational studies. *Br J Nutr.* (2015) 114:1341–59. 10.1017/S0007114515003177 26344504

[13] Harrison S Couture P Lamarche B. Diet quality, saturated fat and metabolic syndrome. *Nutrients.* (2020) 12:3232. 10.3390/nu12113232 33105691 PMC7690379

[14] Martínez-González M Martín-Calvo N. The major European dietary patterns and metabolic syndrome. *Rev Endocr Metabol Dis.* (2013) 14:265–71. 10.1007/s11154-013-9264-6 23979531

[15] Luan D Wang D Campos H Baylin A. Red meat consumption and metabolic syndrome in the costa rica heart study. *Eur J Nutr.* (2020) 59:185–93. 10.1007/s00394-019-01898-6 30649594

[16] Tian Y Su L Wang J Duan X Jiang X. Fruit and vegetable consumption and risk of the metabolic syndrome:a meta-analysis. *Publ Health Nutr.* (2018) 21:756–65. 10.1017/S136898001700310X 29151369 PMC10260986

[17] McCullough M Feskanich D Stampfer M Giovannucci E Rimm E Hu F Diet quality and major chronic disease risk in men and women:moving toward improved dietary guidance. *Am J Clin Nutr.* (2002) 76:1261–71. 10.1093/ajcn/76.6.1261 12450892

[18] Shan Z Li Y Baden M Bhupathiraju S Wang D Sun Q Association between healthy eating patterns and risk of cardiovascular disease. *JAMA Int Med.* (2020) 180:1090–100. 10.1001/jamainternmed.2020.2176 32539102 PMC7296454

[19] Harmon B Boushey C Shvetsov Y Ettienne R Reedy J Wilkens L Associations of key diet-quality indexes with mortality in the multiethnic cohort:the dietary patterns methods project. *Am J Clin Nutr.* (2015) 101:587–97. 10.3945/ajcn.114.090688 25733644 PMC4340063

[20] Bohn T Samouda H Alkerwi A. Dietary patterns and type 2 diabetes—relationship to metabolic syndrome and inflammation. In: Hébert J Hofseth L editors. *Diet, inflammation, and health.* (Chap. 7), Cambridge, MA: Academic Press (2022). p. 261–366. 10.1016/B978-0-12-822130-3.00014-4

[21] Trichopoulou A Costacou T Bamia C Trichopoulos D. Adherence to a Mediterranean diet and survival in a greek population. *New Engl J Med.* (2003) 348:2599–608. 10.1056/NEJMoa025039 12826634

[22] Rosato V Temple N La Vecchia C Castellan G Tavani A Guercio V. Mediterranean diet and cardiovascular disease:a systematic review and meta-analysis of observational studies. *Eur J Nutr.* (2019) 58:173–91. 10.1007/s00394-017-1582-0 29177567

[23] García-Montero C Fraile-Martínez O Gómez-Lahoz A Pekarek L Castellanos A Noguerales-Fraguas F Nutritional components in western diet versus mediterranean diet at the gut microbiota–immune system interplay. implications for health and disease. *Nutrients.* (2021) 13:699. 10.3390/nu13020699 33671569 PMC7927055

[24] Merra G Noce A Marrone G Cintoni M Tarsitano M Capacci A Influence of Mediterranean diet on human gut microbiota. *Kompass Nutr Diet.* (2022) 2:19–25. 10.1159/000523727 PMC7822000 33375042

[25] Asghari G Mirmiran P Yuzbashian E Azizi F. A systematic review of diet quality indices in relation to obesity. *Br J Nutr.* (2017) 117:1055–65. 10.1017/S0007114517000915 28478768

[26] Alkerwi A Bahi I Stranges S Beissel J Delagardelle C Noppe S Geographic variations in cardiometabolic risk factors in Luxembourg. *Int J Environm Res Publ Health.* (2017) 14:648. 10.3390/ijerph14060648 28621751 PMC5486334

[27] Vahid F Brito A Le Coroller G Vaillant M Samouda H Bohn T Dietary intake of adult residents in Luxembourg taking part in two cross-sectional studies&mdash;ORISCAV-LUX (2007-2008) and ORISCAV-LUX 2 (2016-2017). *Nutrients.* (2021) 13:4382. 10.3390/nu13124382 34959934 PMC8706514

[28] Alkerwi A Pastore J Sauvageot N Coroller G Bocquet V d’Incau M Challenges and benefits of integrating diverse sampling strategies in the observation of cardiovascular risk factors (ORISCAV-LUX 2) study. *BMC Med Res Methodol.* (2019) 19:27. 10.1186/s12874-019-0669-0 PMC6360765 30717671

[29] Alkerwi A Pastore J Sauvageot N Coroller G Bocquet V D’Incau M Challenges and benefits of integrating diverse sampling strategies in the observation of cardiovascular risk factors (ORISCAV-LUX 2) study. *BMC Med Res Methdol.* (2019) 19:1–10. 30717671 10.1186/s12874-019-0669-0 PMC6360765

[30] French Agency for Food EaOHS,. *ANSES-CIQUAL French Food Composition Table for Nutritional Intakes Calculation CALNUT.* Available online at: https://ciqual.anses.fr/.

[31] Shah S Novak S Stapleton L. Evaluation and comparison of models of metabolic syndrome using confirmatory factor analysis. *Eur J Epidemiol.* (2006) 21:343–9. 10.1007/s10654-006-9004-2 16736276

[32] Sebekova K Sebek J. Continuous metabolic syndrome score (siMS) enables quantification of severity of cardiometabolic affliction in individuals not presenting with metabolic syndrome. *Bratislavské Lekárske Listy.* (2018) 119:675–8. 10.4149/BLL_2018_121 30672711

[33] Trichopoulou A Kouris-Blazos A Wahlqvist M Gnardellis C Lagiou P Polychronopoulos E Diet and overall survival in elderly people. *BMJ.* (1995) 311:1457–60. 10.1136/bmj.311.7018.1457 8520331 PMC2543726

[34] Bakaloudi D Chrysoula L Kotzakioulafi E Theodoridis X Chourdakis M. Impact of the level of adherence to Mediterranean diet on the parameters of metabolic syndrome:a systematic review and meta-analysis of observational studies. *Nutrients.* (2021) 13:1514. 10.3390/nu13051514 33946280 PMC8146502

[35] Nani A Murtaza B Sayed Khan A Khan N Hichami A. Antioxidant and anti-inflammatory potential of polyphenols contained in Mediterranean diet in obesity:molecular mechanisms. *Molecules.* (2021) 26:985. 10.3390/molecules26040985 33673390 PMC7918790

[36] Wang D Nguyen L Li Y Yan Y Ma W Rinott E The gut microbiome modulates the protective association between a Mediterranean diet and cardiometabolic disease risk. *Nature Med.* (2021) 27:333–43. 10.1038/s41591-020-01223-3 33574608 PMC8186452

[37] Garcia-Lorda P Megias Rangil I Salas-Salvado J. Nut consumption, body weight and insulin resistance. *Eur J clin Nutr.* (2003) 57:S8–11. 10.1038/sj.ejcn.1601802 12947444

[38] Koebnick C Black M Wu J Shu Y MacKay A Watanabe R A diet high in sugar-sweetened beverage and low in fruits and vegetables is associated with adiposity and a pro-inflammatory adipokine profile. *Br J Nutr.* (2018) 120:1230–9. 10.1017/S0007114518002726 30375290 PMC6261668

[39] MacDonald IA. A review of recent evidence relating to sugars, insulin resistance and diabetes. *Eur J Nutr.* (2016) 55:17–23. 10.1007/s00394-016-1340-8 27882410 PMC5174139

[40] Sun S Anderson G Flickinger B Williamson-Hughes P Empie M. Fructose and non-fructose sugar intakes in the US population and their associations with indicators of metabolic syndrome. *Food Chem Toxicol.* (2011) 49:2875–82. 10.1016/j.fct.2011.07.068 21889564

[41] Guo H Ding J Liang J Zhang Y. Association of red meat and poultry consumption with the risk of metabolic syndrome:a meta-analysis of prospective cohort studies. *Front Nutr.* (2021) 8:691848. 10.3389/fnut.2021.691848 34307439 PMC8295459

[42] Hosseini M Sarrafzadegan N Kelishadi R Monajemi M Asgary S Vardanjani H. Population-based metabolic syndrome risk score and its determinants:the Isfahan healthy heart program. *J Res Med Sci.* (2014) 19:1167. PMC4333526 25709659

[43] Cowell O Mistry N Deighton K Matu J Griffiths A Minihane A Effects of a mediterranean diet on blood pressure:a systematic review and meta-analysis of randomized controlled trials and observational studies. *J Hypert.* (2021) 39:729–39. 10.1097/HJH.0000000000002667 33060448

[44] Filippou C Thomopoulos C Kouremeti M Sotiropoulou L Nihoyannopoulos P Tousoulis D Mediterranean diet and blood pressure reduction in adults with and without hypertension:a systematic review and meta-analysis of randomized controlled trials. *Clinical Nutr.* (2021) 40:3191–200. 10.1016/j.clnu.2021.01.030 33581952

[45] Medina-Remón A Tresserra-Rimbau A Pons A Tur J Martorell M Ros E Effects of total dietary polyphenols on plasma nitric oxide and blood pressure in a high cardiovascular risk cohort. the PREDIMED randomized trial. *Nutr Metab Card Dis.* (2015) 25:60–7. 10.1016/j.numecd.2014.09.001 25315667

[46] Bohn T. Carotenoids and markers of oxidative stress in human observational studies and intervention trials:implications for chronic diseases. *Antioxidants.* (2019) 8:179. 10.3390/antiox8060179 31213029 PMC6616644

[47] Upadhyay S Dixit M. Role of polyphenols and other phytochemicals on molecular signaling. *Ox Med Cell Longev.* (2015) 2015:504253. 10.1155/2015/504253 26180591 PMC4477245

[48] De Pergola G D’Alessandro A. Influence of mediterranean diet on blood pressure. *Nutrients.* (2018) 10:1700. 10.3390/nu10111700 30405063 PMC6266047

[49] Thomopoulos C Parati G Zanchetti A. Effects of blood pressure lowering on outcome incidence in hypertension:7. effects of more vs. less intensive blood pressure lowering and different achieved blood pressure levels–updated overview and meta-analyses of randomized trials. *J Hypert.* (2016) 34:613–22. 10.1097/HJH.0000000000000881 26848994

[50] Grao-Cruces E Varela L Martin M Bermudez B Montserrat-de la Paz S. High-density lipoproteins and mediterranean diet:a systematic review. *Nutrients.* (2021) 13:955. 10.3390/nu13030955 33809504 PMC7999874

[51] Shah S Karam J Zeb A Ullah R Shah A Haq I Movement is improvement:the therapeutic effects of exercise and general physical activity on glycemic control in patients with type 2 diabetes mellitus:a systematic review and meta-analysis of randomized controlled trials. *Diabetes Ther.* (2021) 12:707–32. 10.1007/s13300-021-01005-1 33547579 PMC7947168

[52] Fallaize R Livingstone K Celis-Morales C Macready A San-Cristobal R Navas-Carretero S Association between diet-quality scores, adiposity, total cholesterol and markers of nutritional status in European adults:findings from the Food4Me study. *Nutrients.* (2018) 10:49. 10.3390/nu10010049 29316612 PMC5793277

[53] Saraf-Bank S Haghighatdoost F Esmaillzadeh A Larijani B Azadbakht L. Adherence to healthy eating index-2010 is inversely associated with metabolic syndrome and its features among Iranian adult women. *Eur J Clin Nutr.* (2017) 71:425–30. 10.1038/ejcn.2016.173 27677367

[54] Alhazmi A Stojanovski E McEvoy M Garg M. The association between dietary patterns and type 2 diabetes:a systematic review and meta-analysis of cohort studies. *J Hum Nutr Diet.* (2014) 27:251–60. 10.1111/jhn.12139 24102939

[55] Jannasch F Kröger J Schulze M. Dietary patterns and type 2 diabetes:a systematic literature review and meta-analysis of prospective studies. *J Nutr.* (2017) 147:1174–82. 10.3945/jn.116.242552 28424256

[56] Streppel M Arends L van ’t Veer P Grobbee D Geleijnse J. Dietary fiber and blood pressure:a meta-analysis of randomized placebo-controlled trials. *Arch Int Med.* (2005) 165:150–6. 10.1001/archinte.165.2.150 15668359

[57] Siri-Tarino P Chiu S Bergeron N Krauss R. Saturated fats versus polyunsaturated fats versus carbohydrates for cardiovascular disease prevention and treatment. *Ann Rev Nutr.* (2015) 35:517. 10.1146/annurev-nutr-071714-034449 26185980 PMC4744652

[58] de Camargo Talon L de Oliveira E Moreto F Portero-McLellan K Burini R. Omega-3 fatty acids supplementation decreases metabolic syndrome prevalence after lifestyle modification program. *J Funct Foods.* (2015) 19:922–8. 10.1016/j.jff.2015.01.022

[59] Hoffman R Gerber M. Evaluating and adapting the Mediterranean diet for non-Mediterranean populations:a critical appraisal. *Nutr Rev.* (2013) 71:573–84. 10.1111/nure.12040 24032362

