# Supplementary material for: High adherence to the Mediterranean diet and Alternative Healthy Eating Index are associated with reduced odds of metabolic syndrome and its components in participants of the ORISCAV-LUX2 study
Source: Front Nutr. 2022 Dec 13;9:1087985. doi: 10.3389/fnut.2022.1087985 (PMC9793091; doi:10.3389/fnut.2022.1087985)
Supplement: Supplementary file 1 [file Table_1.docx]

Supplementary Material

| **Supplementary Table 1.** The National Cholesterol Education Program Adult Treatment Panel III (NCEP ATP III) definition for metabolic syndrome. | | |
| --- | --- | --- |
| **Risk factors** | **Cut-off points** | **And/or drug treatment** |
| Abdominal obesity (waist circumference) | ≥ 102 cm in men  ≥ 88 cm in women | - |
| Fasting blood glucose | ≥ 100 mg/dL | use of glucose-lowering medication |
| Systolic blood and/or  Diastolic blood pressure | ≥ 130 mmHg  ≥ 85 mmHg | use of antihypertensive medication in a patient with a prior history of hypertension |
| Fasting triglycerides | ≥ 150 mg/dL | use of drug treatment of elevated triglycerides fibrates and/or nicotinic acid |
| High-density lipoprotein cholesterol | < 40 mg/dL (1.04 mmol/L) in men  < 50 mg/dL (1.3 mmol/L) in women | use of drug treatment for low HDL-C, fibrates, and/or nicotinic acid |
|  |  |  |

| **Supplementary Table 2.** The scoring system for the Alternate Healthy Eating Index (AHEI) food groups. Total scoring ranges from 0 to 75 points. | |
| --- | --- |
| **Components** | **Scores** |
| Vegetables | ­+10 points for 5 servings/day  0 points for no servings/day |
| Fruits | +10 points for 4 servings/day  0 points for no servings/day |
| Replacement of white for red meat | +10 points for a 4:1 ratio  0 points for less  *Vegetarians received a score of 10 |
| Nuts and soy protein | +10 points for 1 serving/day  0 points for no servings/day |
| Cereal fiber | +10 points for 15+g/day  0 points for no g/day |
| The ratio of polyunsaturated to saturated fat | +10 points for a ratio ≥ 1  0 point for ˂0.1 |
| Multivitamin supplements | +5 points for consuming any supplement  0 point for all others |
| Moderate alcohol consumption | +10 points for 1.5-2.5 servings/day men  +10 points for 0.5-1.5 servings/day women  0 point for no consumption  0 point for ˃3.5 servings/day men  0 point for ˃2.5 servings/day women |

| Supplementary Table 3. The association of the dietary indices MDS and AHEI with MetS components, as derived by linear regression (beta non-standardized, 95% CI, p-value**, and strength of linear association R). | | | | | | | | | | | | | | | |
| --- | --- | --- | --- | --- | --- | --- | --- | --- | --- | --- | --- | --- | --- | --- | --- |
| Dependent^a^/  Independent variables | **Model-A** | | | | | **Model-B** | | | | | **Model-C Full** | | | | |
|  | **Beta** | **95% CI** | | **P-value** | **R** | **Beta** | **95% CI** | | **P-value** | **R** | **Beta** | **95% CI** | | **P-value** | **R** |
| BMI*/MDS Quartiles | -0.0069 | -0.0104 | -0.0035 | **<0.001** | 0.552 | -0.0063 | -0.0096 | -0.0030 | **<0.001** | 0.307 | -0.0087 | -0.0128 | -0.0045 | **<0.001** | 0.353 |
| WC/MDS Quartiles | -0.0062 | -0.0091 | -0.0033 | **<0.001** | 0.111 | -0.0049 | -0.0074 | -0.0024 | **<0.001** | 0.516 | -0.0059 | -0.0090 | -0.0028 | **<0.001** | 0.558 |
| FBG/MDS Quartiles | -0.0025 | -0.0054 | 0.0004 | 0.090 | 0.046 | -0.0022 | -0.0049 | 0.0004 | 0.101 | 0.384 | -0.0008 | -0.0037 | 0.0021 | 0.586 | 0.427 |
| TG/MDS Quartiles | -0.0121 | -0.0221 | -0.0019 | **0.015** | 0.066 | -0.0087 | -0.0179 | 0.0005 | 0.064 | 0.334 | -0.0035 | -0.0152 | 0.0080 | 0.546 | 0.396 |
| HDL/MDS Quartiles | 0.0023 | -0.0027 | 0.0074 | 0.359 | 0.025 | -0.0014 | -0.0059 | 0.0031 | 0.537 | 0.476 | -0.0005 | -0.0061 | 0.0050 | 0.844 | 0.536 |
| SBP/MDS Quartiles | -0.0050 | -0.0077 | -0.0024 | **<0.001** | 0.099 | -0.0044 | -0.0067 | -0.0021 | **<0.001** | 0.512 | -0.0055 | -0.0084 | -0.0026 | **<0.001** | 0.532 |
| DBP/MDS Quartiles | -0.0050 | -0.0077 | -0.0022 | **<0.001** | 0.096 | -0.0045 | -0.0071 | -0.0019 | **0.001** | 0.336 | -0.0041 | -0.0075 | -0.0008 | **0.015** | 0.342 |
|  |  |  |  |  |  |  |  |  |  |  |  |  |  |  |  |
| BMI*/AHEI Quartiles | -0.0011 | -0.0041 | 0.0003 | 0.768 | 0.008 | -0.0021 | -0.0048 | 0.0021 | 0.326 | 0.293 | -0.0068 | -0.0121 | -0.0018 | **0.007** | 0.354 |
| WC/AHEI Quartiles | 0.0019 | -0.0006 | 0.0048 | 0.180 | 0.036 | -0.0001 | -0.0019 | 0.0029 | 0.791 | 0.508 | -0.0048 | -0.0089 | -0.0009 | **0.015** | 0.556 |
| FBG/AHEI Quartiles | -0.0012 | -0.0043 | 0.0003 | 0.680 | 0.011 | -0.0018 | -0.0048 | 0.0009 | 0.186 | 0.384 | -0.0019 | -0.0054 | 0.0016 | 0.286 | 0.428 |
| TG/AHEI Quartiles | -0.0030 | -0.0130 | 0.0068 | 0.545 | 0.016 | -0.0066 | -0.0160 | 0.0027 | 0.166 | 0.333 | -0.0026 | -0.0164 | 0.0111 | 0.710 | 0.396 |
| HDL/AHEI Quartiles | -0.0043 | -0.0095 | 0.0008 | 0.098 | 0.045 | -0.0023 | -0.0069 | 0.0022 | 0.307 | 0.477 | -0.0018 | -0.0084 | 0.0047 | 0.587 | 0.537 |
| SBP/AHEI Quartiles | -0.0005 | -0.0033 | 0.0021 | 0.675 | 0.011 | -0.0021 | -0.0044 | 0.0002 | 0.079 | 0.506 | -0.0040 | -0.0074 | -0.0005 | **0.021** | 0.525 |
| DBP/AHEI Quartiles | -0.0021 | -0.0049 | 0.0006 | 0.131 | 0.040 | -0.0032 | -0.0058 | -0.0005 | **0.018** | 0.330 | -0.0033 | -0.0072 | 0.0006 | 0.099 | 0.337 |
| ^a^ All dependent variables were entered in the models as log-transformed.  Model A: the dependent variable was one of the components, and the independent variable was one of the indices as quartiles.  Model B: the dependent variable was one of the components, and the independent variables were one of the indices as quartiles, in addition to age groups and gender as confounding factors.  Model C: the dependent variable was one of the components, and the independent variable was one of the indices as quartiles, and the confounding factors were age group, gender, and all sociodemographic variables and selected anthropometric variables (education, job, income, marital status, and country of birth, physical activity, currently smoking, and total energy intake).  AHEI: alternative healthy eating index, MDS: Mediterranean diet score, BMI: Body Mass Index, WC: Waist circumference, FBG: Fasting blood glucose levels, TG: Triglycerides, HDL: High-density lipoprotein cholesterol, SBP: Systolic blood pressure, DBP: Diastolic blood pressure.  * The BMI is not a component of the MetS and is additionally analyzed for additional information.  ** Significant values are given in bold. | | | | | | | | | | | | | | | |

| Supplementary Table 4. The association between MetS and dietary indices MDS and AHEI is determined by (bivariate) logistic regression expressed with odd ratio (OR), p-value, and 95% CI (lower bound-upper bound). | | | | |
| --- | --- | --- | --- | --- |
| Indices | **P-value** | **OR** | **95% CI** | |
|  | **Model-A** | | | |
| MDS | 0.273 | 0.956 | 0.882 | 1.036 |
| MDS Quartiles | 0.369 | 0.953 | 0.858 | 1.058 |
| AHEI | 0.967 | 1.000 | 0.988 | 1.012 |
| AHEI Quartiles | 0.821 | 0.988 | 0.887 | 1.100 |
|  | **Model-B** | | | |
| MDS | 0.152 | 0.937 | 0.858 | 1.024 |
| MDS Quartiles | 0.243 | 0.934 | 0.833 | 1.047 |
| AHEI | 0.495 | 0.995 | 0.982 | 1.009 |
| AHEI Quartiles | 0.410 | 0.952 | 0.846 | 1.070 |
|  | **Model-C** | | | |
| MDS | 0.234 | 0.925 | 0.813 | 1.052 |
| MDS Quartiles | 0.308 | 0.916 | 0.774 | 1.084 |
| AHEI | 0.810 | 0.997 | 0.974 | 1.021 |
| AHEI Quartiles | 0.956 | 0.994 | 0.813 | 1.217 |
| Model A: the dependent variable was MetS as a categorical variable, and the independent variable is one of the indices (AHEI score, AHEI quartiles, MDS score, MDS quartiles).  Model B: the dependent variable is metabolic syndrome, and the independent variables were one of the indices with confounding factors of age group and gender.  Model C: the dependent variable was metabolic syndrome, and the independent variables were one of the indices with confounding factors, which were age group, gender, and all sociodemographic variables and selected anthropometric variables such as (education, job, income, marital status, country of birth, physical activity, currently smoking, total energy intake). | | | | |

| **Supplementary Table 5.** The association of the MetS scores with MDS and AHEI, as calculated by linear regression (beta non-standardized, 95% CI, p-value*, and strength of linear association R). | | | | | | |
| --- | --- | --- | --- | --- | --- | --- |
| **Models** | **Dependent^a^/Independent variables** | **Beta** | **P-value** | **95%CI** | | **R** |
| Model A |  |  |  |  | |  |
|  | SIMS/MDS Quartile | -0.064 | **0.020** | -0.277 | -0.024 | 0.064 |
|  | SIMS/AHEI Quartile | -0.020 | 0.455 | -0.179 | 0.080 | 0.020 |
|  | EFA/MDS Quartile | -0.132 | **<0.001** | -0.491 | -0.209 | 0.132 |
|  | EFA/AHEI Quartile | -0.041 | 0.130 | -0.257 | 0.033 | 0.041 |
|  | MetSR/MDS Quartile | -0.092 | **<0.001** | -0.418 | -0.112 | 0.092 |
|  | MetSR/AHEI Quartile | 0.034 | 0.219 | -0.059 | 0.255 | 0.034 |
| Model B |  |  |  |  |  |  |
|  | SIMS/MDS Quartile | -0.049 | 0.063 | -0.238 | 0.006 | 0.277 |
|  | SIMS/AHEI Quartile | -0.037 | 0.158 | -0.214 | 0.035 | 0.275 |
|  | EFA/MDS Quartile | -0.126 | **<0.001** | -0.457 | -0.213 | 0.519 |
|  | EFA/AHEI Quartile | -0.071 | **0.003** | -0.317 | -0.067 | 0.508 |
|  | MetSR/MDS Quartile | -0.055 | **<0.001** | -0.249 | -0.069 | 0.814 |
|  | MetSR/AHEI Quartile | -0.016 | 0.302 | -0.140 | 0.043 | 0.812 |
| Model C |  |  |  |  |  |  |
|  | SIMS/MDS Quartile | -0.031 | 0.330 | -0.236 | 0.079 | 0.384 |
|  | SIMS/AHEI Quartile | -0.034 | 0.365 | -0.273 | 0.100 | 0.384 |
|  | EFA/MDS Quartile | -0.119 | **<0.001** | -0.452 | -0.159 | 0.534 |
|  | EFA/AHEI Quartile | -0.106 | **0.002** | -0.452 | -0.103 | 0.529 |
|  | MetSR/MDS Quartile | -0.054 | **0.003** | -0.257 | -0.052 | 0.848 |
|  | MetSR/AHEI Quartile | -0.040 | 0.064 | -0.238 | 0.007 | 0.847 |
| ^a^ All dependent variables were entered in the models as log-transformed.  Model A: the dependent variable was one of the MetS Score, and the independent variable was one of the indices, as quartiles.  Model B: the dependent variable was one of the MetS Score, and the independent variables were one of the indices as quartiles, in addition to age groups and gender as confounding factors.  Model C: the dependent variable was one of the MetS Score, and the independent variable was one of the indices as quartiles, and the confounding factors were age group, gender, and all sociodemographic variables and selected anthropometric variables (education, job, income, marital status, and country of birth, physical activity, currently smoking, and total energy intake).  *Significant values are given in **bold.** | | | | | | |
